# Evidence of international transmission of mobile colistin resistant monophasic *Salmonella* Typhimurium ST34

**DOI:** 10.1038/s41598-023-34242-4

**Published:** 2023-05-01

**Authors:** Sirirak Supa-amornkul, Rattanaporn Intuy, Wuthiwat Ruangchai, Soraya Chaturongakul, Prasit Palittapongarnpim

**Affiliations:** 1 Mahidol International Dental School, Faculty of Dentistry, Mahidol University, Bangkok, Thailand; 2 Department of Microbiology, Faculty of Science, Pornchai Matangkasombut Center for Microbial Genomics, Mahidol University, Bangkok, Thailand; 3 Molecular Medical Biosciences Cluster, Institute of Molecular Biosciences, Mahidol University, Nakhon Pathom, Thailand; 4 Department of Microbiology, Faculty of Science, Mahidol University, Rama 6 Road, Bangkok, 10400 Thailand

**Keywords:** Genetics, Microbiology

## Abstract

*S.* 4,[5],12:i:-, a monophasic variant of *S. enterica* serovar Typhimurium, is an important multidrug resistant serovar. Strains of colistin-resistant *S.* 4,[5],12:i:- have been reported in several countries with patients occasionally had recent histories of travels to Southeast Asia. In the study herein, we investigated the genomes of *S.* 4,[5],12:i:- carrying mobile colistin resistance (*mcr*) gene in Thailand. Three isolates of *mcr-3.1* carrying *S.* 4,[5],12:i:- in Thailand were sequenced by both Illumina and Oxford Nanopore platforms and we analyzed the sequences together with the whole genome sequences of other *mcr-3* carrying *S.* 4,[5],12:i:- isolates available in the NCBI Pathogen Detection database. Three hundred sixty-nine core genome SNVs were identified from 27 isolates, compared to the *S.* Typhimurium LT2 reference genome. A maximum-likelihood phylogenetic tree was constructed and revealed that the samples could be divided into three clades, which correlated with the profiles of *fljAB-hin* deletions and plasmids. A couple of isolates from Denmark had the genetic profiles similar to Thai isolates, and were from the patients who had traveled to Thailand. Complete genome assembly of the three isolates revealed the insertion of a copy of IS*26* at the same site near *iroB,* suggesting that the insertion was an initial step for the deletions of *fljAB-hin* regions, the hallmark of the 4,[5],12:i:- serovar. Six types of plasmid replicons were identified with the majority being IncA/C. The coexistence of *mcr-3.1* and *bla*_*CTX-M-55*_ was found in both hybrid-assembled IncA/C plasmids but not in IncHI2 plasmid. This study revealed possible transmission links between colistin resistant *S.* 4,[5],12:i:- isolates found in Thailand and Denmark and confirmed the important role of plasmids in transferring multidrug resistance.

## Introduction

Multidrug-resistant *Salmonella enterica* serotype 4,[5],12:i:-, a monophasic variant of *S. enterica* serovar Typhimurium, has become more prevalent and widespread in the last two decades. Colistin is the last resort antibiotic against the multidrug-resistant bacteria. Recently, mobile colistin resistance (*mcr*) genes have been identified in many species of *Enterobacteriaceae* family including *Salmonella enterica*, particularly *S.* 4,[5],12:i:-^1^. Reports from England^2^, Denmark^3^, Canada^4^, and USA^5^ revealed that several infected patients had traveled to China or Southeast Asian countries, including Thailand. This suggested a circulation of the colistin resistant *S.* 4,[5],12,i:- strains in the region, which might occasionally be transmitted internationally.

There are 10 *mcr* genes, designated as *mcr-1* to *mcr-10*, all encoding phosphoethanolamine transferases, which modify lipid A at the outer membrane of Gram-negative bacteria. Several *mcr* genes, including *mcr-1*, -*3*, -*6*, -*7*, -*8*, and -*9* have been reported in *Escherichia coli* in Thailand with *mcr-1* being the most common, followed by *mcr-9*^6^. In addition to *E. coli*^7,8^ *mcr-1* was also reported from several other species of *Enterobacteriaceae* in Thailand^9,10^ including *Klebsiella*^11^, *Edwardsiella*^12^ and recently *S. enterica* serovar Cannstatt^13^. *mcr-3* has been reported in *Aeromonas veronii*^14^, *E. coli*^15^, *K. pneumoniae*^13,16^, *Enterobacter*^17^ and recently *S. enterica* serovar 4,[5],12:i:-^18–22^.*mcr-3* shares 45% homology to *mcr-1*. It is usually located upstream of diacylglycerol kinase gene (*dgkA*) and flanked by a truncated and intact IS*Kpn40*. The ΔIS*Kpn40-mcr3-dgkA-*IS*kpn40* segment is flanked by IS*26* and IS*15DI* in IncHI2 or pWJ1 plasmid^23^. In IncA/C2 or IncHI2A/IncY plasmid, the fragment is flanked by two IS*15DI* elements^2^.

We screened for the presence of the *mcr* genes in whole genome sequences (WGS) of 53 isolates of *S.* 4,[5],12,i:- in Thailand and identified three isolates with *mcr-3* but none of the other *mcr* genes. In order to gain more understanding on the transmission dynamics of *mcr-3* mediated resistance, we compared the genomes of these three isolates with the available genomic sequences of other *mcr-3*-carrying isolates of *S.* 4,[5],12,i:- deposited in public databases. The analysis revealed that *mcr-3* genes were carried by several plasmids and the similarity of plasmid profiles conformed partially with the core genome phylogeny. The findings conformed to the notion that some European patients might be infected by *S.* 4,[5],12,i:- in Thailand and suggest that the deletion of *fljAB-hin* has been mediated by recombination of IS*26*.

## Results

Three samples of *S.* 4,[5],12:i:-, all isolated in 2010, harbored *mcr-3.1*. Two were from human stool and the other was from ready-to-eat frozen food. Phenotypic characterization confirmed that all three isolates were resistant to colistin, all with MIC and MBC of 8 µg/ml.

WGS of H1-012, H1-014 and H1-120 were analyzed together with the ones of 25 other isolates from several countries. WGS of all 28 isolates covered > 95% of the genome of the LT2 strain, as shown in Supplementary Table 1.

Long read sequence data statistics and the genome metrics of the H1-012, H1-014 and H1-120 are shown in Supplementary Table 2. Their chromosomal sizes ranged from 4,929,877 to 5,039,354 bp, and the GC content was approximately 52.1%.

### Structural variations of the *fljAB-hin* regions

*fljAB-hin* region deletion is a hallmark of *S.* 4,[5],12:i:-. This region is generally replaced by drug resistance genes. We mapped the presence and absence of genes around the *fljAB-hin* region from *STM2692* (*hlyD*) to *STM2775* (*iroD*, enterochelin esterase) by blasting the gene sequences of the *S.* Typhimurium LT2 reference against the de novo assembled contigs of the short read sequences of each *S.* 4,[5],12:i:- isolate. The presence of some genes cannot be ascertained, including *STM2768-STM2769*, both probably encoding IS*3* transposases. Their multiple presence in the genome might preclude accurate mapping. Many genes were clearly absent or present. However, the assembled contigs might also contain only some parts of the genes. We consider the results as ambiguous because the partial absence can be a result of incomplete assembly.

Of 28 isolates, mapping the *fljAB-hin* region of isolate 2008AR-0009 (Genbank Biosample Number SAMN07688908) from USA did not identify any deletions compared to *S*. Typhimurium LT2. Thus, the strain should still be considered as *S.* Typhimurium and was consequently excluded from further analysis. The other 27 isolates all lacked Fels-2 prophage genes (*STM2693* to *STM2739*), present in *S*. Typhimurium LT2. Only *STM2740*, coding for phage integrase protein, consistently remained intact. Eight patterns of deletions were identified with the shortest one involving only *fljAB-hin* deletions while 70 genes were deleted in the longest one as shown in Supplementary Fig. 1. In all 27 isolates, the right borders of the deleted region were at the same position on the 5’ non-coding region of *iroB* side but variable on the other side. Identification of *iroB* revealed ambiguous results in three cases, though.

### Core genome phylogeny, *fljAB-hin* deletion and presence of plasmid replicons.

**Figure 1:**
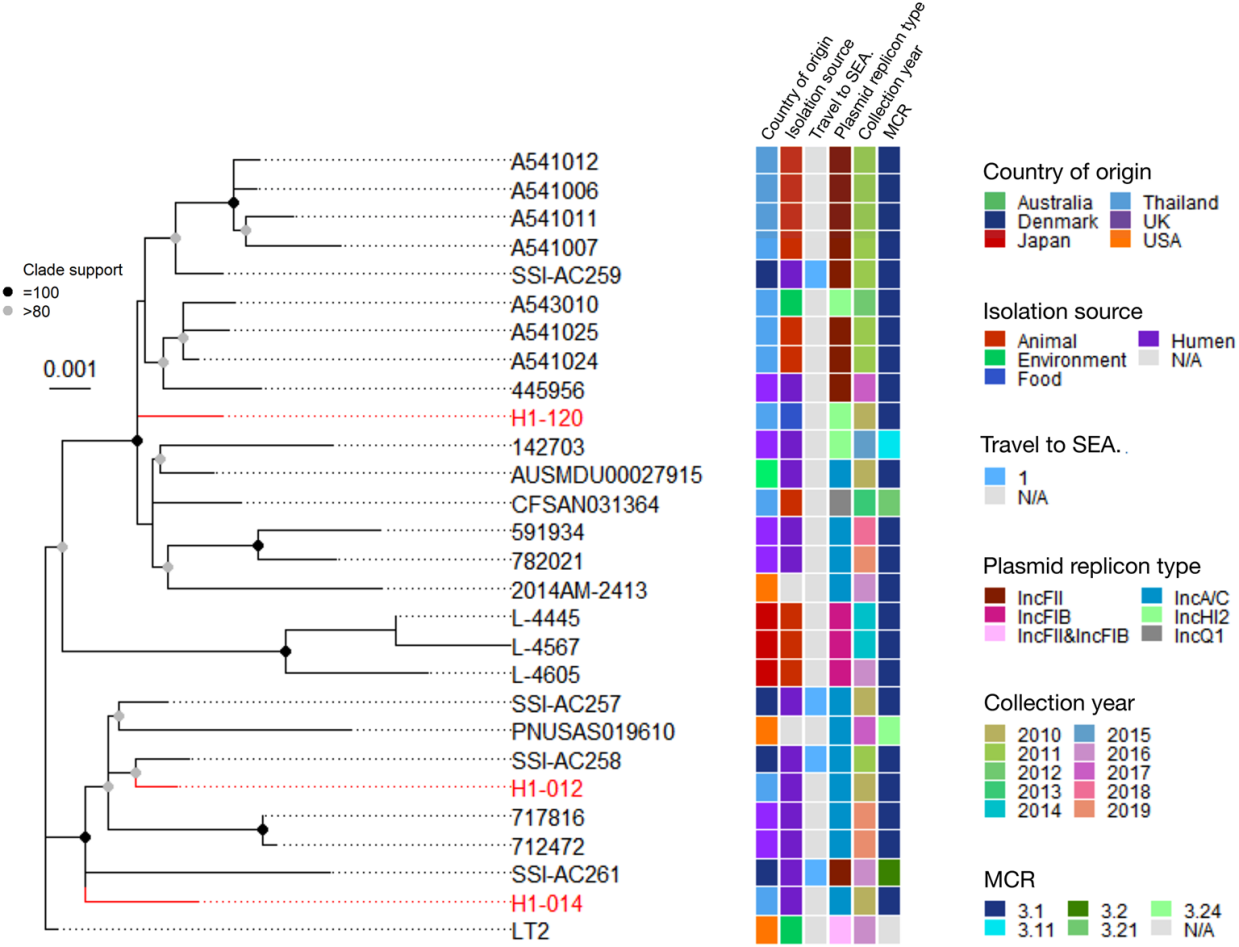
A core genome SNV ML phylogenetic tree of 27 *mcr-3* carrying *S*. 4,[5].12:i:- isolates. The names of the samples sequenced in this study are colored in red. The country of origin, the isolation sources, the existing history of travel to Southeast Asia (SEA), the isolation year and the type of *mcr* are presented by the panel of colored squares on the right. The black circle represents the clade that has the bootstrap value of 100 and the gray circle indicates the bootstrap value of more than 80.

The single nucleotide variants (SNVs) in the core genome of 27 isolates were identified using *S.* Typhimurium LT2 as reference. A total of 369 of core genome SNVs were identified. All isolates belonged to ST34, with the SNV differences from *S*. Typhimurium LT2 ranging from 16–46 SNVs (average 31) as shown in Supplementary Table 3. Their pairwise SNV distances ranged from 2–79 with the average pairwise SNV distance of 45.1 and the median of 45. A maximum-likelihood phylogenetic tree was constructed and shown in Fig. 1. The bootstrap scores were calculated, and the bootstrap score of 100 was used to define a clade. The bacteria were separated into three clades.

The first clade comprised eight isolates from Denmark (3), UK (2), USA (1) and two isolates sequenced here (H1-012 and H1-014). All isolates were from human sources, except for the one from the USA whose source was unknown. Six isolates carried *mcr-3.1* together with IncA/C plasmid replicon while the other two carried *mcr-3.24* with IncA/C and *mcr-3.20* with IncFII and IncX1 (Supplementary Table 4). Mapping the *fljAB* region revealed that all had the deletion of *fljA-fljB* (*STM2770-2771*) and *hin (STM2772)* with the conservation of *iroB* (Supplementary Fig. 1b). However, the 5’ ends of the deletions were distinct except for two pairs of isolates, which also had relatively small SNV distances. The first pair from the UK (712,472 and 717,816) had a pairwise distance of only two and both shared a large deletion from *STM2703* to *hin*. The second pair of isolates from Thailand (H1-012) and Denmark (SSI-AC258) had a pairwise distance of 11. Both isolates similarly had complete absence of only *fljAB* and *hin*. SSI-AC258 might also lose a part of *STM2769* upstream to *fljA*, though. These results indicated that the two UK isolates were epidemiologically linked and the Denmark isolate was probably the same clone as H1-012, which had been isolated a year earlier.

The second clade comprised three Japanese cattle isolates, all carrying IncFIB plasmid. Isolates L-4445 and L-4567 were close to each other, with the pairwise SNV distance being 14 while L-4605 was 26 and 41 SNVs distant from the first pair, respectively (Supplementary Table 3). All three had an identical *fljAB* deletion starting from *STM2753* to *hin*, which was different from all other isolates in this study (Supplementary Fig. 1b).

The third clade included seven isolates from Denmark (1), UK (4), USA (1) and Australia (1), seven isolates from swine or swine production environment in Thailand and the food isolate sequenced here (H1-120) (Fig. 1). Interestingly, in contrast to the first clade, all isolates in this clade shared the same deletion from *STM2760* to *hin* (Supplementary Fig. 1)*.* The early branching isolates in the tree tend to have IncA/C plasmid while eight of the nine isolates in the terminal branch carry IncFII (Fig. 1). The latter included all the seven swine isolates from Thailand, isolated in 2011–2014. They were distributed into two clades with average pairwise SNV distances of 10.2 and 8.33 (Supplementary Table 3). Their average interclade pairwise SNV distance was 23.6. They were probably parts of a bigger outbreak in swine. The terminal branch also included two human IncFII-carrying isolates from Denmark and the UK, isolated in 2011 and 2017 respectively. The Denmark (SSI-AC259) and the UK (445,956) isolates were 13 and 17 SNVs different from the closest swine isolates respectively, which were shorter than the average interclade differences (Supplementary Table 3). SSI-AC259 was isolated from a patient with a travel history to Thailand in 2011^3^, indicating the epidemiological linkage of SSI-AC259 and the swine outbreaks. The UK isolate, isolated much later in 2017, had a different deletion profile upstream the deleted *STM2760*-*hin* segment and was, therefore, more remotely linked to the swine outbreak (Supplementary Fig. 1).

Pangenome analysis revealed a total of 3,063 hard-core and 950 soft-core genes shard by 27 isolates as well as 1,575 accessory genes (Supplementary Fig. 2a). The number of shared genes in each clade is higher than core genes as shown in (Supplementary Fig. 2b–d).

Comparing the *fljAB-hin* deletion patterns with the SNV distances reveals that a pair of isolates with SNVs distance less than 11 always had the same *fljAB-hin* deleted segment while a pair of isolates with SNVs distances more than 55 always had different *fljAB-hin* deletion patterns as shown in Supplementary Fig. 1b. Summary of the *fljAB-hin* deletion patterns of the 27 *mcr-3* carrying *S.* 4,[5],12:i:- isolates is shown in Table 1. Overall, the data indicated that the isolates with genetically related core genomes and similar *fljAB-hin* region deletion profiles were likely to share the same *mcr-3.1* carrying plasmids as illustrated in Fig. 1.

**Table 1:** Summary of the *fljAB-hin* deletion patterns of the 27 *mcr-3* carrying *S.* 4,[5],12:i:- isolates.

Deleted regions	Numbers of isolates	Associated plasmid replicons	Remarks
*STM2760-hin*	16 (Including H1-120)	IncFII, IncC and IncHI2/IncHI2A	All belong to the same clade
*STM2753-hin*	3	IncFIB	All belong to the same clade (Japanese isolates)
*STM2746-hin*	1 (SSI-AC261)	*mcr* 3.20 in IncFII	–
STM2759-hin*	1 (H1-014)	IncA/C	–
*fljAB-hin*	2 (H1-012 and SSI-AC258^#^)	IncA/C	–
*STM2761-hin*	1 (SSI-AC257)	IncA/C	–
*STM2740-hin*	2 (712,472 and 717,816)	IncA/C	A pair of isolates from the UK
*STM2746-hin*	1 (PNUSAS019610)	*mcr*-3.24 in IncA/C	–

* Partial deletion of *STM2759* is confirmed by long read sequencing, ^#^Possible partial deletion of *STM2769*. Table 1 reports the possible associated plasmid replicon types among isolates with different *fljAB-hin* deletion patterns. For example, each of the 16 isolates (including H1-120) with the *STM2760-hin* deletion contains one of the three types of replicons. H1-120 has two contigs, one chromosome and one plasmid.

### *fljAB-hin* regions of H1-012, H1-014 and H1-120

**Figure 2:**
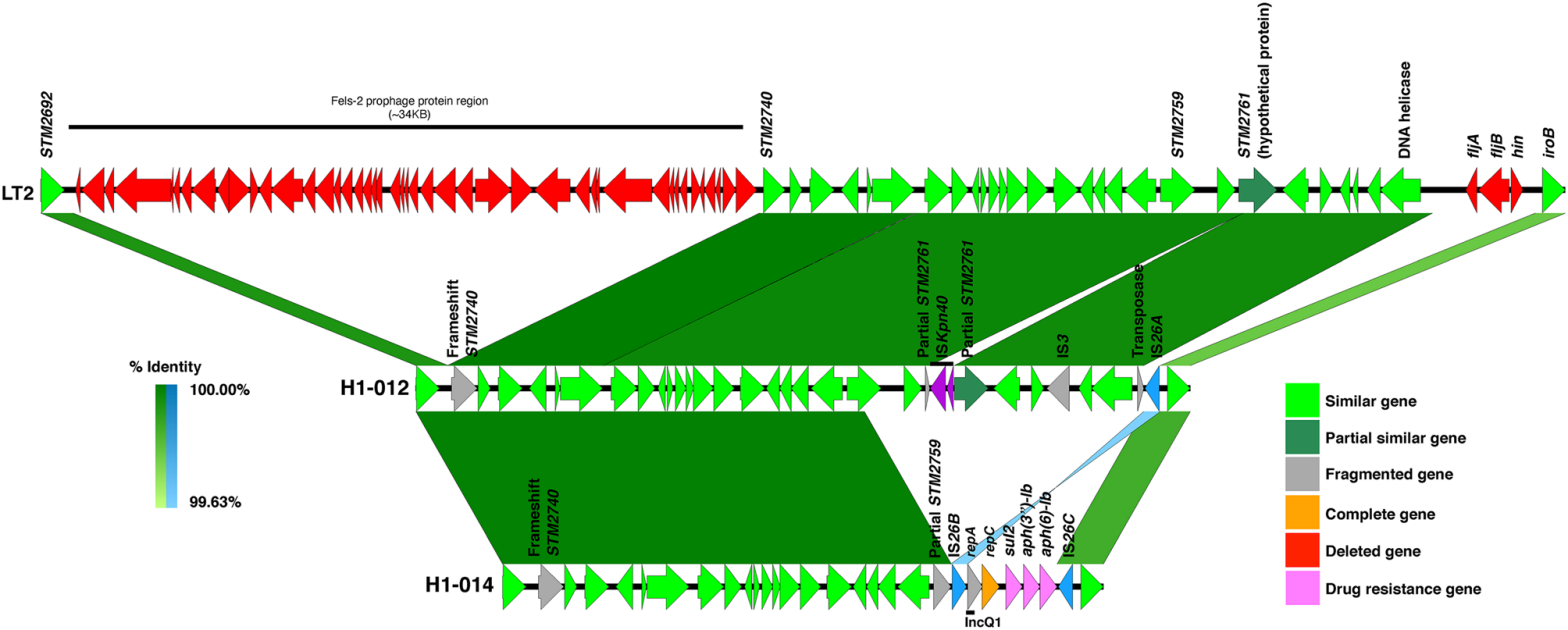
Comparative gene maps of *S*. Typhimurium LT2 (upper), *S*. 4,[5],12:i:- H1-012 (middle) and H1-014 (lower). H1-012 is characterized by deletion of the *fljAB-hin* segment with the replacement of IS*26* and insertion of IS*Kpn40*. H1-014 had a larger deletion and insertion of a few resistance genes, flanked on both ends by IS*26*, the 3’ one exactly at the same position as H1-012.

The *fljAB-hin* deleted regions of H1-012, H1-014 and H1-120 were investigated using the hybrid-assembled sequences. Lack of the Fels-2 prophage (*STM2693* to *STM2739*) was confirmed. The deleted region of H1-012, which was the shortest, was 5053 bp long corresponding to position 2,910,987–2,916,039 in the *S*. Typhimurium LT2 genome, which included only *fljA, fljB* and *hin.* The deleted region was replaced by an 820-bp-long segment carrying a transposase, with a pair of an inverted repeat (GGCACTGTTGCAAA) (Supplementary Fig. 3) at the ends on both sides, identified as IS*26* (IS*6* family). Nevertheless, there was no drug resistance gene inserted in the region (Fig. 2). There was an additional insertion of 1,213-bp-long IS*Kpn40* (IS*3* family) at position 2,901,703 immediately downstream *STM2761*. The copy of IS*Kpn40* was flanked by a direct repeat CCGG.

The deleted region of H1-014 was 17,540 bp long corresponding to the position 2,898,500–2,916,039 in the *S*. Typhimurium LT2 genome, which included 14 genes, starting from nucleotide 772 of *STM2759* (losing 909 nucleotides) to *hin.* The deleted segment was replaced by a 14,524-bp-long common DNA fragment containing *repA, repC, sul2, aph(3″)-Ib, and aph(6)-Id*, flanked by two copies of IS*26* (Fig. 2). The 3’ copy of the IS*26* of H1-014 resided at exactly the same nucleotide position as the one in H1-012. Moreover, a replicon sequence of IncQ1 was identified in the inserted segment.

**Figure 3:**
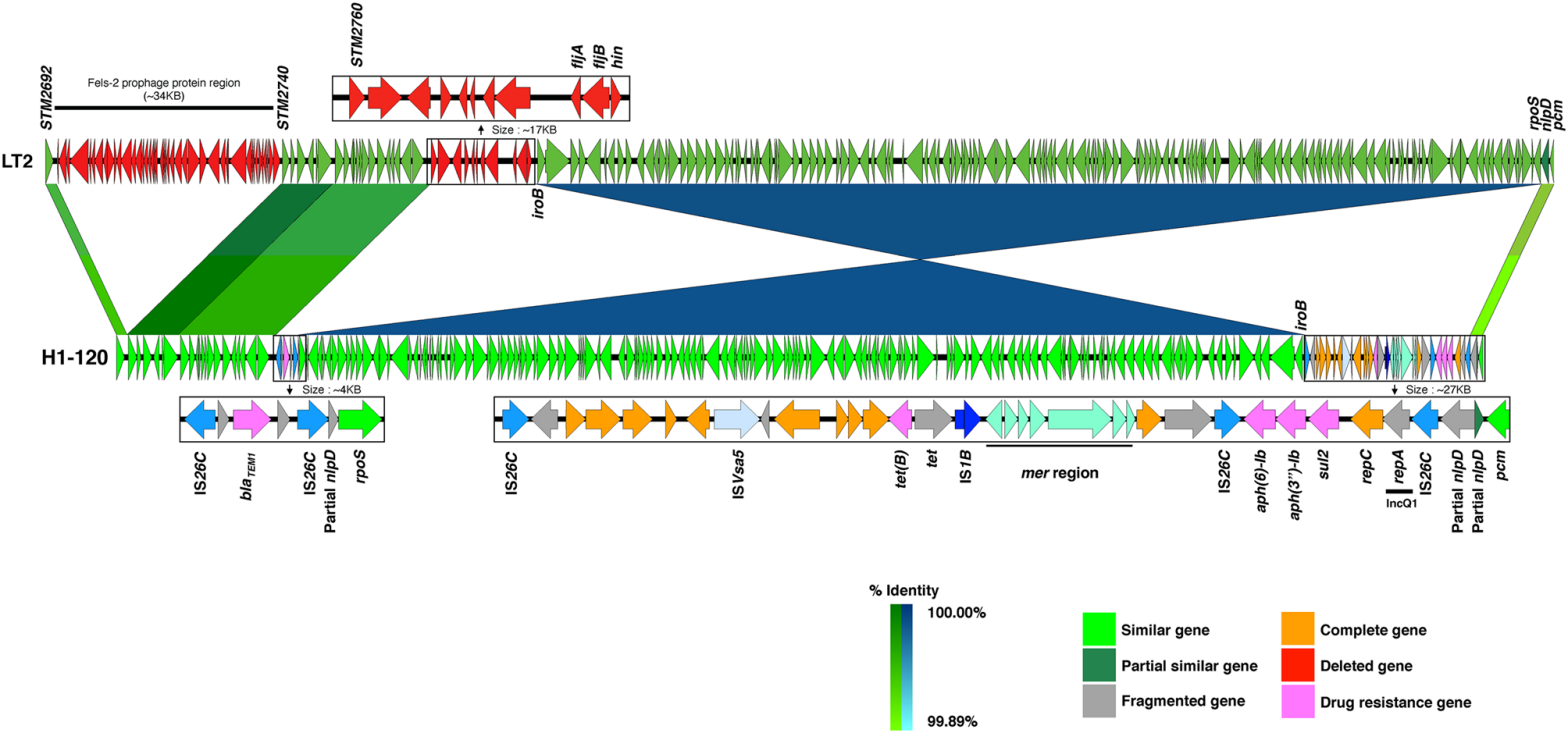
Comparative gene maps of *S*. Typhimurium LT2 and, *S*. 4,[5],12:i:- H1-120. H1-120 is characterized by deletion of the *fljAB-hin* segment and a large inversion of a DNA segment with insertions of two segments of multidrug resistance genes and *mer genes* (lower) which were flanked on both ends by IS*26C.*

The 15,715-bp-long deleted region of H1-120 included 13 genes from *STM2760* to *hin* (position 2,900,325–2,916,039)*,* which is different from H1-014, as it did not include a part of *STM2759.* Interestingly the deleted regions of all three isolates ended at the same nucleotide position. The deletion in H1-120 was accompanied by an inversion of a 150,626-bp-long segment (position 2,916,040–3,066,666), including 152 genes from *STM2773* (*iroB*) to *STM2924* (*rpoS*) and the last 111 bp of *STM2925c* (*nlpD*). The inverted segment was flanked on both sides by IS*26*. There were two inserted resistance gene regions on both sides of the large inverted region, between *STM2759* and the last 111 bp of *STM2925*, as well as between *iroB* and the remaining part of *STM2925* (*nlpD*) as shown in Fig. 3. The former segment contained only *bla*_*TEM-1*_, flanked by two IS*26*. The latter inserted region, also flanked on both sides by IS*26*, comprised two parts, separated by another copy of IS*26*. The major part contained several metabolic genes, two drug resistance genes (*tetR*(B) and *tet*) and a *mer* operon (*merR, merT, merP, merC, merA, merD* and *merE*). The metabolic genes co-integrated with the drug resistance gene included genes encoding DNA (cytosine-5-)-methyltransferase, thermonuclease, phospholipase D, LysR family transcriptional regulator, sodium/glutamate symporter, and antibiotic biosynthesis monooxygenase. The minor part was the *aph(6)-Id*-*aph(3″)-Ib-sul2-repC-repA* segment similar to the one found in H1-014 (Fig. 2).

Comparison of the IS*26* transposase sequences revealed that all copies of IS*26* in H1-120 shared the same sequence, tentatively designated as IS*26C*. So was the 3’ copy of IS*26* in H1-014. They were 2 bp different from the 5’ copy of H1-014 (designated as IS*26B*) and a bp different from the copy in H1-012 (IS*26A*) as shown in Supplementary Fig. 3 and Supplementary Table 5.

### Comparison of plasmid sequences of *mcr-3* carrying *S.* 4,[5],12:i:-

Plasmid replicons were identified from WGS data of all isolates using PlasmidFinder. Three and nine isolates carry plasmids IncFIB and IncFII, respectively. Eleven isolates, including H1-012 and H1-014, carry IncA/C ST3 plasmid while three isolates, including H1-120, carry IncHI2 plasmid (Fig. 1, Supplementary Table 4).

### The plasmid sequences of H1-012, H1-014 and H1-120

To better understand the structural variation of *mcr-3* carrying plasmids as well as their contributions to the spread of drug resistance, circular plasmid contigs were constructed from the combined Illumina/Nanopore sequencing data. The plasmids in H1-012, H1-014 and H1-120 were 180,560 bp, 179,719 bp and 222,107 bp long, respectively, and designated as pH1-012, pH1-014 and pH1-120.

**Figure 4:**
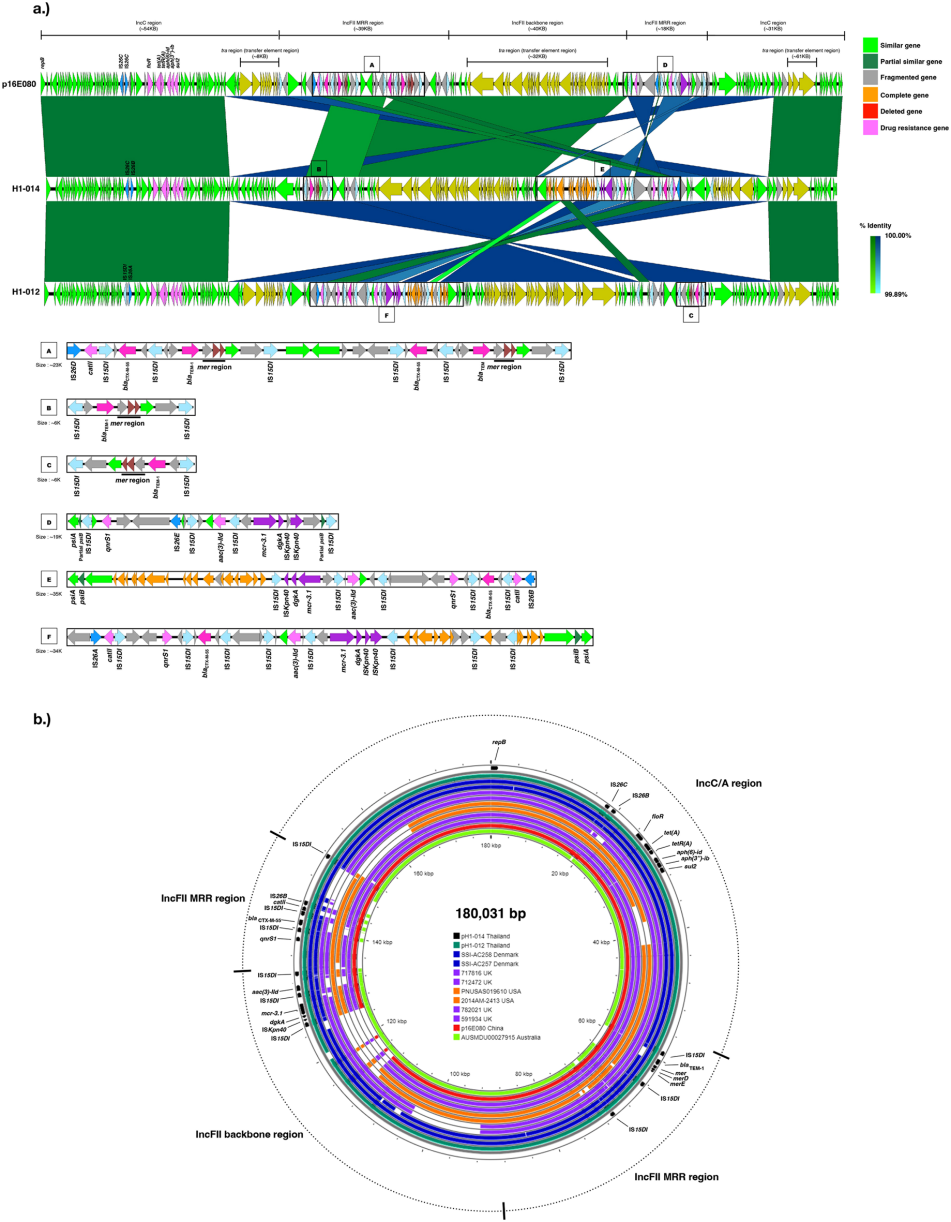
Plasmid alignment of p16E080, pH1-014 and pH1-012. (**a**) Linear gene maps of p16E080, pH1-014 and pH1-012 plasmids starting from *repB.* The complete sequences of pH1-012 and pH1-014 were from the hybrid assemblies. The gene arrangement in the multidrug resistance and *mcr* regions were enlarged. (**b**) Short read mapping of other IncA/C carrying isolates in this study, using pH1-014 as reference. pH1-012 and the plasmid of SSI-AC258 were very similar.

The complete sequences of pH1-012 and pH1-014 were compared with the reference IncA/C-FII ST3 plasmid p16E080^24^ (180 kb, GenBank accession number: MN647788) isolated from a human sample in China in 2016 (Fig. 4). All three plasmids had comparable length and actually were hybrids of IncA/C and IncFII.

Using the *repB* as the first gene of the plasmids, all three plasmids contained two major parts. The segments on both sides of the *repB* genes (position 1–41,449 and 164,501–179,719 in pH1-014), which were parts of the original IncC sequence, were fairly conserved and had the same orientation. These segments contained three small drug resistance gene clusters namely, *floR, tet(A)-tetR(A)* and *aph(6)-ld–aph(3″)-Ib*–*sul2*.

The remaining parts of pH1-012 and pH1-014, which include the IncFII sequence segment and parts of IncC, were inverted while the orientation of the IncFII segment in H1-014 and p16E080 were the same. There were many small in/del, transposition and inversion, mainly in the drug resistance gene regions. The drug resistance genes in the region of all three plasmids were clustered into two subregions. The first subregions of H1-012 and H1-014 contain *bla*_*TEM-1*_*, merD* and *merE* flanked on both sides by IS*15DI*. Interestingly, the same region in p16E080 also contained *bla*_*CTX-M-55*_ in tandem to the *bla*_*TEM-1*_ segment and the entire set was duplicated in the same orientation.

The second subregions contained the *mcr-3.1*, accompanied by *dgkA*, IS*Kpn40* and flanked by IS*15DI* on both sides similar to the previous report^2^. There was *aac(3)-IId* and *qnrS1* upstream to *mcr-3.1* in all three plasmids. Interestingly, in H1-012 and H1-014, there is also *bla*_*CTX-M-55*_ downstream to *qnrS1*. However, their orientations of *qnrS1-bla*_*CTX-M-55*_ were inverted relative to *mcr-3.1*. Further upstream of *mcr-3.1*, there is *catII* in both H1-012 and H1-014 but not in p16E080. *pslB* is located distantly downstream of *mcr-3.1* in both H1-012 and H1-014, with partially similar intervening sequences. However, *pslB* in p16E080 was split and present on both sides of this subregion. In short, pH1-012 and pH1-014 were similar albeit non-identical plasmids while p16E080 was more distantly related. *bla*_*CTX-M-55*_ was closer to the *mcr-3.1* genes in pH1-012 and pH1-014.

**Figure 5:**
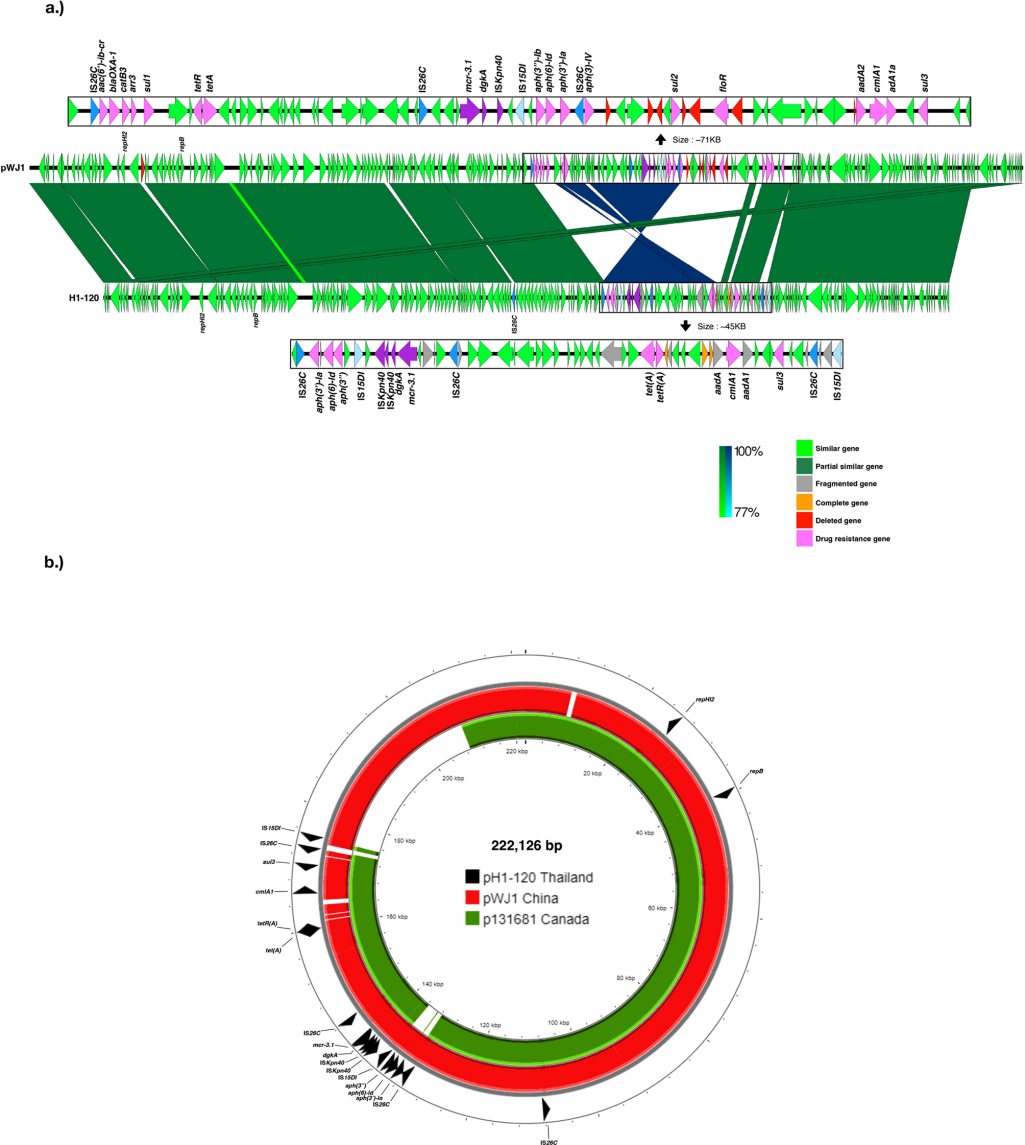
Plasmid alignment of pWJ1and pH1-120. (**a**) Linear gene maps of pWJ1and pH1-120 plasmids. pWJ1 was used as a reference for IncHI2 plasmids. Gene arrangement in multidrug resistance and *mcr* regions were enlarged. The order of genes is according to pWJ1 [23]. (**b**) Circular alignment of IncHI2 plasmids of pH1-120, p131681 and pWJ1. pH1-120 was used as a reference for short read mapping of other isolates.

The sequence of pH1-120 was compared to the reference pWJ1 plasmid, an IncHI2 plasmid, firstly reported as a *mcr-3* containing plasmid from *E. coli* in China^23^. pH1-120 was shorter than pWJ1 with most genes in the same orientation (Fig. 5). The shorter pH1-120 was associated with the lack of two drug resistance gene regions (*aac(6’)-lb-cr*, *bla*_*OXA-1*_, *catB3*, *arr3* and *sul1*; and, *sul2* and *floR*) and a 14,095-bp-long segment which were duplicated in pWJ1 and had the same sequence as position 93 -14,032 in pH1-120. However, *sul2* was present in the chromosome of H1-120 isolate (Figs. 3 and 5a).

The genes surrounding *mcr-3.1* in the IncHI2 plasmid were different from those of IncA/C plasmid. The *mcr-3.1* was accompanied with *dgkA,* Is*Kpn40* and flanked by IS*26* and IS*15DI* (Fig. 5a), similar to the previous report^23^.

IS*26* plays an important role in disseminating antibiotic resistance genes. Three more variants of IS*26* were identified in the plasmid, including IS*15DI*, which is only a bp different from IS*26*B. The other two were designated as IS*26D* and IS*26E*. The sequence relationship of all IS*26* was summarized in Supplementary Fig. 4. IS*15DI* was present in all analyzed plasmids, particularly IncA/C (Figs. 4,5) but absent from *fljAB-hin* regions. IS*26C* was present not only in *fljAB-hin* region and pH1-014 and pH1-120 but also in p16E080 and pWJ1*.* IS*26C* has the same sequence as IS*26* in *Proteus vulgaris* (Genbank accession number X00011.1).

To further explore the similarity between the plasmids, the short reads of the other IncA/C carrying isolates as well as p16E080 were mapped to the pH1-014 (Fig. 4b). The method did not allow the evaluation of the gene orders. All the IncA/C plasmids contained both the IncA/C and IncFII parts, similar to pH1-014. pH1-012 and the plasmid of SSI-AC258 were very similar. The *mcr-3.1* and *dgkA*, flanked by IS*15DI* were present in all IncA/C plasmids, presumably in the IncFII part similar to pH1-014. The other antibiotic resistance genes present in all plasmids included *qnrS1* and a group of antibiotic resistance genes composed of *floR, tet(A), tetR(A), aph(6)-Id, aph(3″)-Ib* and *sul2* (Fig. 4b)*. bla*_*CTX-M-55*_ and *bla*_*TEM-1*_ was present in 9 and 10 of the 12 plasmids respectively. The possible coexistence of *mcr-3.1* and *bla*_*CTX-M-55*_ in the same plasmid conformed to the observations of the association between colistin resistance and extended spectrum beta-lactamase (ESBL)^19^.

In contrast to the isolates carrying IncFII, the ones carrying IncA/C had a variety of *fljAB* deletions, including four isolates with *fljAB* deletion similar to the ones carrying IncFII (Supplementary Fig. 1). This might signify the spreading potential of the IncA/C plasmids across various variant strains of *S.* 4,[5],12:i:-. Our results from de novo hybrid assembly of pH1-012 and pH1-014 show that in our IncA/C plasmids contain the replicon of IncFII. Both IncA/C and IncFII replicons contain their own *tra* region (Fig. 4a) which make the IncA/C plasmids have two sets of the *tra* regions. These might increase the chance of horizontal transmission.

Three isolates carried IncHI2 but did not form a clade in the phylogenetic tree. H1-120 carries a multi-replicon plasmid IncHI2/IncHI2A, belonging to ST3, similar to the plasmid pWJ1 (261 Kb, GenBank accession number KY924928^23^), originally identified as *mcr-3* carrying plasmid in *E. coli* [23] (Fig. 5). A different IncHI2/IncHI2A plasmid, belonging to ST2, was identified to carry *mcr-3.11* in an isolate from the UK (142,703) (Supplementary Table 4). The last isolate carrying only IncHI2 was from a swine in Thailand (A543010) and is phylogenetically close to the IncFII carrying isolates. Figure 5b shows short read mapping by using H1-120 as a reference. The isolates UK 142,703 (UK) and A543010 (Thailand) are not included in the figure because there belonged to different ST and had different plasmid replicon. p131681 (202 kb) was isolated from a patient in Canada, in April 2013^4^. He had traveled to Thailand a month prior to the isolation of the bacterium. p131681 was include in the figure because it was identified as IncHI2/IncHI2A plasmid, ST3. From mapping, p131681 is the smallest plasmid.

Fourteen isolates carried IncQ1 replicon. IncQ1 is a small plasmid which has never been reported to carry *mcr-3*. As the IncQ1 replicon is identified in the chromosomes of H1-014 and H1-120, it is not clear whether there really was the IncQ1 plasmid in the other isolates. Nevertheless, it should be noted that there was an *mcr-3* carrying isolate that did not carry any other plasmids apart from IncQ1.

### Antimicrobial resistance (AMR) gene search

The AMR genes of H1-012, H1-014 and H1-120 were identified by ResFinder and found in both chromosomes and plasmids. Twenty antibiotic resistance genes (6 genes in the chromosomes and 18 genes in the plasmids) related to resistance to eight antibiotic classes were found in the isolates. Five and three genes were related to resistance to aminoglycosides beta-lactams (*aac(6’)-Iaa*, *aac(3)-Iid, aph(3’)-Ia, aph(3’’)-Ib* and *aph(6)-Id*) and beta-lactamase (*bla*_*CTX-M-55*_, *bla*_*TEM-1B*_, and *bla*_*TEM-216*_.) respectively. Most antibiotic resistance genes in H1-012 (10 genes), H1-014 (9 genes) and H1-120 (11 genes) were found more in the plasmids than in the chromosomes, where there were 1, 5 and 6 genes, respectively (Supplementary Table 6). Most of antibiotic resistance genes in the chromosome were found in the *fljAB-hin* deletion region. Only *aac(6')-Iaa* was found outside the region in all three isolates, between genes coding for Doer/GlpR family DNA-binding transcription and lactate oxidase.

We compared the antibiotic resistance phenotype (AMRP) and predicted antibiotic resistance phenotype (PAMRP) from ResFinder. The result shows that all AMRP of the three isolates agreed with PAMRP. (Supplementary Table 7).

## Discussion

*S.* 4,[5],12,:i:- is multidrug resistant with increasing report of colistin resistance^1,19^. Here we reported the complete genomes of three colistin resistant *S.* 4,[5],12:i:- and investigated their core genome phylogeny, *fljAB-hin* deletion profiles and plasmids in comparison with WGS of 24 other isolates of *mcr-3* carrying *S.* 4,[5],12,:i:- deposited in NCBI. All isolates belonged to ST34 with the differences of the core genomes not more than 71 SNVs, but had variable *fljAB-hin* deletions. The similarity of the deletion profiles and plasmids correlated with the shorter SNV distances between isolates. This suggests that combination of the information helps recognizing epidemiologically linked isolates.

Transmission of *S.* 4,[5],12:i:- is evidenced by the presence of a few pairs or groups of isolates with a small numbers of SNV differences, the identical *fljAB-hin* deletions and the identical *mcr-3* carrying plasmids replicon types, indicating epidemiological linkages within each pair or group. The bacterial isolates of a number of patients in developed countries who had a history of travel to Thailand were closely related to isolates from swine or food suggesting the transmission mechanism as food-borne^24^.

This study illustrates the usefulness of open genomic repository for identifying possible transmission routes of *Salmonella*. In order to do so, a SNV difference cutoff may be needed to infer which pairs of isolates were epidemiologically related. Various cutoffs for *Salmonella enterica* were proposed^25^. A previous study on outbreaks of *S.* Typhimurium in Australia revealed that most of the outbreak isolates had not more than two SNV differences^26^. This is a very strict criterion compared to other bacteria or other serovars of *Salmonella* and probably suitable for local outbreaks but not for identification of international transmission. The study here indicated that a more relaxed SNP cutoff criterion of 11 may be useful for screening for possibly epidemiological linkage of *S.* 4,[5],12,:i:- pairs. The suspected linkage can be further strengthened by the similarity of the *fljAB-hin* deletion profiles and plasmid replicon types. In reverse, the fact that core-genome related isolates can harbor different plasmids as shown here demonstrates both the plasticity of the plasmids as vehicles for transferring drug resistance as well as the limitation of the plasmid profiling for inferring transmission.

*S*. 4,[5],12:i:- is characterized by the deletion of the *fljAB*-*hin* region, usually replaced by drug resistance gene cassettes. Nevertheless, the complete genome sequence of H1-012 demonstrated that the replacement by drug resistance genes is not mandatory. The replacement of *fljAB-hin* of H1-012 by a single copy of IS*26*, which is in the same genomic position as the other sequenced isolates supported the previous suggestion that the IS*26* transposition was an initial step for the development of the monophasic *S*. Typhimurium^27^. The fact that the IS*26* in H1-012 inserted at the same nucleotide position as the 3’ copy of IS*26* in H1-014 and H1-120 indicates that they descended from an ancestor harboring that particular copy.

The absence of identical short direct repeat at both ends of the IS*26* in H1-012 suggests that the copy was a result of recombination of two original IS*26* on both sides of the *fljAB*-*hin* region. We hypothesize that the variation of *fljAB-hin* deletions are the results of the insertion of the second copy of IS*26* at different positions upstream of the *fljAB* resulted in the deletions of different sizes after homologous recombination. The multiple events of IS*26* recombination resulting in the deletion of *fljAB-hin* suggest an evolutionary benefit for the loss of the *fljAB-hin* genes, which results in the loss of phase 2 flagella. The appearance of the antibiotic resistance genes was likely to be a result of subsequent recombination with a resistance gene cassette flanked by IS*26*.

Colistin resistance is a serious problem^28^. A recent study indicated that the majority of *mcr* in *Salmonella* was *mcr-3*, which was found in 4 of 26 investigated *S*. 4,[5],12:i:- in Thailand^19^. It was also reported that *mcr-3* sometimes co-propagate with an ESBL *bla*_CTX-M-55_^19^. Here we confirmed that both genes co-existed in the IncFII part of the same conjugable IncA/C-FII hybrid plasmid. This is worrisome as the propagation of both important resistance genes may occur easily among many enteric bacterial species.

The fact that isolates that had closer core genome phylogenetic relationship and similar *fljAB-hin* deletion profiles tended to carry similar plasmids suggests that the spread of mobile colistin resistance in *S.* 4,[5],12:i:- is more attributable to the spread of the host strains than by the spread of plasmids. Nevertheless, several plasmid replicons involved in the transmission of *mcr-3.1* warrant further surveillance of the plasmids in other Enterobacteriaceae.

It should be noted that the first reported plasmid replicon carrying *mcr-3.1*, IncHI2, contributed only to the minority of isolates in this study. In contrast to *mcr-1*, the data here, therefore, do not support the hypothesis that IncHI2 might be a more efficient vessel to disseminate *mcr* genes than other plasmids^29^.

In addition to IncHI2/IncHI2A, originally reported to carry *mcr-3.1*, three other plasmids namely IncC, IncFII, and IncFIB were also implicated. Interestingly, *mcr-3* has not been reported from other common Enterobacteriaceae such as *E. coli* or *Klebsiella* in Thailand where the *mcr* genes in *E. coli* are carried mostly by IncX4 and IncI2^13^. The origins of the *mcr-3* carrying plasmids in *Salmonella* in Thailand are not clear and remain to be further studied.

## Conclusion

This study provided the evidences supporting the international transmission of *mcr-3*-carrying isolates of *S.* 4,[5],12:i:-. This demonstrates the benefits of a global genomic database of *Salmonella*, which would be an important tool for identifying and hopefully preventing the international transmission of *Salmonella*. In this study we also found *mcr-3* in IncA/C plasmid more often than the other groups of plasmids which is different from *mcr-1* gene.

The complete genome information suggests the important role of IS*26*, including the closely related IS*15DI*, in the antibiotic resistance gene exchange among bacterial chromosomes and plasmids, as suggested from the distribution of multiple copies of IS*26* in the area around multidrug resistance genes. Many inversions of gene-order were observed due to the presence of insertion sequences suggesting that only data from short read sequencing might not be optimal to study the evolution of drug resistance genes.

## Materials and methods

### Samples

Twenty-two human isolates and 31 non-human isolates of Thai *S.* 4,[5],12,i:- collected during 2009–2012 were previously sequenced^30^. The presence of *mcr* genes were identified using ResFinder^31^. Among all *mcr* genes, only *mcr-3* was identified in three isolates i.e., H1-012, H1-014 and H1-120. The three samples were additionally sequenced by Oxford Nanopore.

We also included available short read data of *mcr-3* harboring S. 4,5,[12]:i:- isolates that were already published before 2020 or available in the Pathogen Detection database (https://www.ncbi.nlm.nih.gov/pathogens/). Seven isolates were from swine farms in Northern Thailand^32^. The origins of the other samples were Australia (1), Denmark (4), Japan (3), Thailand (1), United Kingdom (6) and USA (3). The information regarding the samples was shown in Supplementary Table 8.

### Phenotypic and genetic profiles of the three isolates

H1-012 and H1-014 isolates were isolated from humans in 2010 and had similar antimicrobial resistance (AMR) patterns i.e., resistant to ampicillin (Amp), cefotaxime (Ctx), chloramphenicol (C), ciprofloxacin (Cp), streptomycin (S) and tetracycline (T)^33^. H1-120 was isolated from frozen food in 2010 and demonstrated resistance to Amp, C, S, T, and sulfamethoxazole/trimethoprim (Sxt)^33^. Genetic studies of the three isolates were previously reported^30,33^. Their PCR patterns indicated the absence of *fljAB*-*hin* region. The PFGE patterns of H1-012 and H1-014 were the same but different from that of H1-120^33^.

### Antibiotic susceptibility testing by microdilution method

Colistin resistance phenotypes in the three isolates were confirmed by determining the minimum inhibitory concentration (MIC) and minimum bactericidal concentration (MBC) using a broth microdilution method in cation-adjusted Muller Hinton broth (CA-MHB). Eight concentrations (0.5, 1, 2, 4, 8, 16, 32 and 64 µg/ml) of colistin were used^34^.

### WGS analysis

#### Short read sequencing

The short read sequencing was previously done^30^. The accession number and the numbers of reads of all selected isolates were shown in Supplementary Table 8.

For long read sequencing, *Salmonella* isolates were cultured in Trypticase Soy broth (TSB) at 37 °C for 16 h. Genomic DNA was extracted using the QIAmp DNA mini kit (Qiagen, Hilden, Germany). The DNA quality and quantities were assessed with Nanodrop DenoVix® and the Qubit 3.0 fluorometer. The total input of DNA was about 400 ng for each flow cell. Separation and determination of the sizes of linear DNA fragments were carried out by electrophoresis through submerged 1.0% horizontal agarose gels.

The genomic DNA was ligated according to the manufacturer’s instructions. Libraries were sequenced with qualified FLO-MIN106 flow cells (R9.4.1, active pore number > 800) for approximately 72 h on MinION (Oxford Nanopore Technologies, Oxford, UK). Base-calling was performed in real time using Guppy with a base calling model modified for 6 mA dam/5 mC dcm and CpG, which was integrated in the MinKNOW software v3.5.40 installed on MinION. The long read sequencing data of H1-012, H1-014, and H1-120 are under BioProject accession numbers PRJNA675488 and PRJNA808666.

### SNV calling and core genome phylogeny

The fastq files of all 27 isolates were quality-trimmed using Trimmomatic v0.39^35^ with the following parameters: sliding-window trimming with a window size of 4 with read quality threshold of 30 and minimum read length of 70 bp. The remaining high-quality reads were mapped to the complete genome of *S*. Typhimurium LT2 (GenBank accession number: NC_003197.2) using BWA program^36^ with the mem algorithm, skipping seed (-c) 100, marking split hits as secondary mapping (-M), and only reporting read with a minimum 50 score (-T 50). Per-sample single nucleotide variants (SNVs) were called using GATK HaplotypeCaller v4.1.6.0^37^ with a base quality score >  = 20 and haploid model. Joint SNV calling of all 27 isolates was performed using GATK GenotypeGVCFs v4.1.6.0. A single nucleotide variant (SNV) alignment was made and filtered by using *VariantFiltration* and *SelectVariants* functions (GATK) with quality by depth (QD) >  = 2 or root mean square of mapping quality (MQ) >  = 40. The SNV positions having less than 50% calling bases from the whole population were excluded. This resulted in 1273 SNVs across 27 genomes.

The SNV alignment was filtered with the core genome position list^38^ resulting in 490 SNV sites. Potential recombinant regions within the alignment were checked using Recombination Detection Program 4^39^. The recombination regions detected by more than four programs were excluded. Three hundred and sixty-nine nucleotides remained after the removal of the recombination regions.

A maximum-likelihood (ML) phylogenetic tree of 27 isolates was inferred using IQ-TREE v2^40^ with ultrafast bootstrap supports from 1000 replications. The best-fit nucleotide model was determined to be K3P + ASC + R2 under the Bayesian information criterion by ModelFinder^41^. The *S*. Typhimurium LT2 (GenBank accession number: NC_003197.2) was used as an outgroup for rooting the tree, which was visualized with Figtree v1.43 (http://tree.bio.ed.ac.uk/software/figtree/). Pairwise SNV distances were calculated using MEGA7^42^.

### Short read assembly

De novo short read assembly was performed using Unicycler v.0.4.8.0^43^. The Illumina short reads were quality checked using FastQC v0.11.9^44^ and trimmed using Trimmomatic v.0.38^35^ with average quality per read >  = 30.

### Hybrid de novo assembly and annotation

De novo hybrid assembly was done using the approach of Unicycler v0.4.8.0^43^. The raw reads of Illumina were quality checked using FastQC v0.11.9^44^ and then trimmed using Trimmomatic v0.38.0^35^. The mean read quality of the raw long reads of MinION was scored using Nanoplot v1.0.0^45^. The adapters on the end of the raw reads were trimmed using Porechop v0.2.4^46^. Trimmed reads with the minimum length of 1000 bp were used for subsequent assembly using Filtlong V.0.2.0^47^. The complete genomes were annotated using Prokaryotic Genome Annotation Pipeline (PGAP) by NCBI^48^. The de novo hybrid assembly of each isolate resulted in two circular contigs: a chromosome and a plasmid.

### Plasmid identification and comparison

Plasmid replicon types were analyzed using PlasmidFinder v2.1 online tool^49–51^. Plasmid comparisons were performed using Easyfig 2.2.5 program^52^.

### Genome identification and drug-resistance detection

Multi-locus sequence typing (MLST) and plasmid MLST (pMLST) were identified using PubMLST online tools^53^ with *Salmonella spp*. as the interested organisms and the plasmid MLST database. Antibiotic resistance genes were detected using ResFinder v4.1 online tool^31,50,51,54^ with *Salmonella spp*. as the interested organisms.

### Pangenome

The analysis included a total of 27 isolates. Sequence data of isolates H-012, H-014, and H-120 were from hybrid de novo assembly results and only represent chromosomal DNA. The sequence data of the Japan isolates (L-4445, L-4567, and L-4605) were from the NCBI Rep-Seq database and, again, represent only chromosomal DNA. The remaining 21 isolates were obtained from short-read de novo assembly and represent both chromosomal and plasmid DNA. Contigs from the de novo short-read assembly that were less than 1,000 bp in length were excluded from the analysis. To perform the pan-genome analysis, we utilized the Panaroo program version 1.3.2 with strict mode^55^.

## Supplementary Information

Supplementary Information.

## Data Availability

The long read sequencing data of H1-012, H1-014 and H1-120 are available in the Sequence Read Archive repository, under BioProject accession number PRJNA675488 for H1-012 and H1-014 and PRJNA808666 for H1-120.
