# Supplementary material for: Evidence of international transmission of mobile colistin resistant monophasic Salmonella Typhimurium ST34
Source: Sci Rep. 2023 May 1;13:7080. doi: 10.1038/s41598-023-34242-4 (PMC10151351; doi:10.1038/s41598-023-34242-4)
Supplement: Supplementary file 1 — Supplementary Information. [file 41598_2023_34242_MOESM1_ESM.docx]

**Additional data**

**Title**: Evidence of International Transmission of Mobile Colistin Resistant Monophasic *Salmonella* Typhimurium ST34.

Sirirak Supa-amornkul^1,2^, Rattanaporn Intuy^2^, Wuthiwat Ruangchai^2^, Soraya Chaturongakul^2,3^, Prasit Palittapongarnpim^2,4*^.

^1^ Mahidol International Dental School, Faculty of Dentistry, Mahidol University, Thailand

^2^ Pornchai Matangkasombut Center for Microbial Genomics, Department of Microbiology, Faculty of Science, Mahidol University, Thailand

^3^ Molecular Medical Biosciences Cluster, Institute of Molecular Biosciences, Mahidol University, Thailand

^4^ Department of Microbiology, Faculty of Science, Mahidol University, Thailand

*Corresponding author: Prasit Palittapongarnpim, Department of Microbiology, Faculty of Science, Mahidol University, Rama 6 Road, Bangkok 10400, Thailand. prasit.pal@mahidol.ac.th

Submit to Scientific Reports

**Contents**

1. **Supplementary Figure 1** a.) Phylogenetic tree of the studied isolates constructed from the SNVs in the core genome.
2. **Supplementary Figure 2** Pangenome analysis of 27 S. 4,[5],12:i:- isolates .
3. **Supplementary Figure 3** Alignment of H1-012, H1-014, and H1-120 in the region near *iroB* using *S.* Typhimurium LT2 as a reference.
4. **Supplementary Figure 4** Minimum spanning tree of various alleles of IS*26* in this study and in *Proteus vulgaris* (accession number X00011.1).
5. **Supplementary Table 1** Short read mapping and SNV calling statistics of 27 *S*. 4,[5],12:i:- isolates with *S.* Typhimurium LT2 as a reference genome
6. **Supplementary Table 2** The long read sequence data statistics and characteristics of the complete genome sequences
7. **Supplementary Table 3** Pairwise SNV distances of 27 *mcr-3* carrying *S.* 4,[5],12:i:- and *S.* Typhimurium LT2
8. **Supplementary Table 4** Plasmid information of all isolates in this study.
9. **Supplementary Table 5** Pairwise SNV distances of IS*26* alleles found in this study compared to the ones in *Proteus vulgaris*. (Accession number X00011.1).
10. **Supplementary Table 6** AMR genes in each antibiotic class found in H1-012, H1-014 and H1-120 complete genomes as identified by ResFinder^3^
11. **Supplementary Table 7** Comparison between the antibiotic resistance phenotype (AMRP) and the predicted antibiotic resistance phenotype by ResFinder (PAMRP) of in H1-012, H1-014, and H1-120
12. **Supplementary Table** 8 Isolates information and demographic information.
13. **References**

**
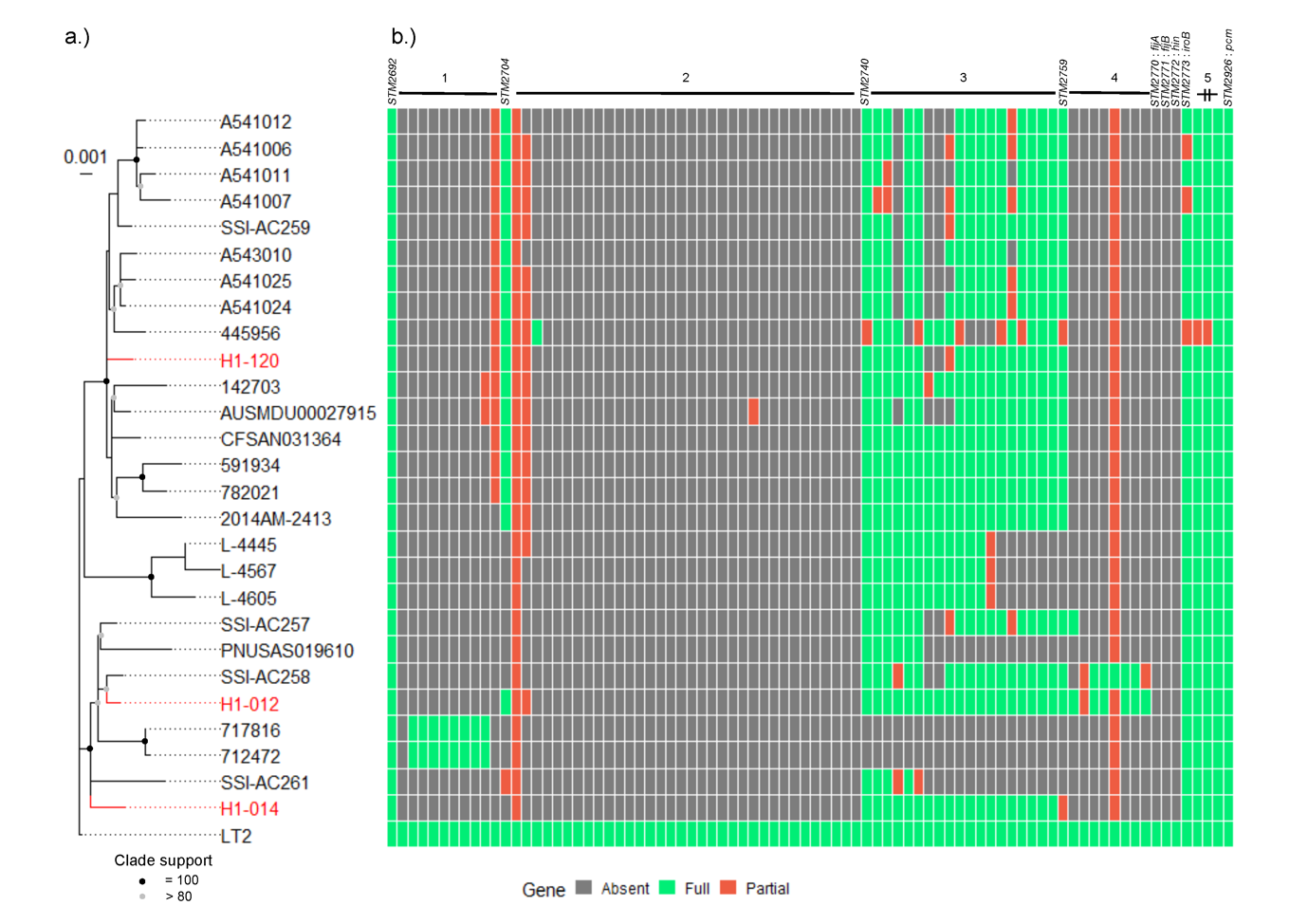
**

**Supplementary Figure 1** a.) Phylogenetic tree of the studied isolates constructed from the SNVs in the core genome. The black circles represent the clades that have the bootstrap values of 100 and the gray circles indicate the bootstrap values of more than 80 b.) The presence (green) and the absence (dark gray) of *STM2692* to *STM2926* in 27 *mcr-3* carrying *S.* 4,[5],12:i:- isolates were determined by mapping their short reads to the *S.* Typhimurium LT2 reference genome. The red indicates partial presence, which is considered as ambiguous. Region 1 contains *STM2694* to *STM2703.* Region 2 contains *STM2705* to *STM 27039.*  Region 3 contains *STM2741* to *STM 2758.* Region 4 contains *STM2760* to *STM 2767.* Finally, Region 5 contains *STM2774* to *STM 2925.* The genes *STM2693* (regulatory RNA 10Sa)-*STM2740* (phage integrase protein) belonged to Fels-2 prophage. The longest contiguous deletions involving *fljAB-hin* in isolates 717816 and 712472 ranged from STM2702 to STM 2772. However, the mapping was not able to map highly paralogous genes, such as transposases. This resulted in the number of genes smaller than 70 shown in the figure.

**
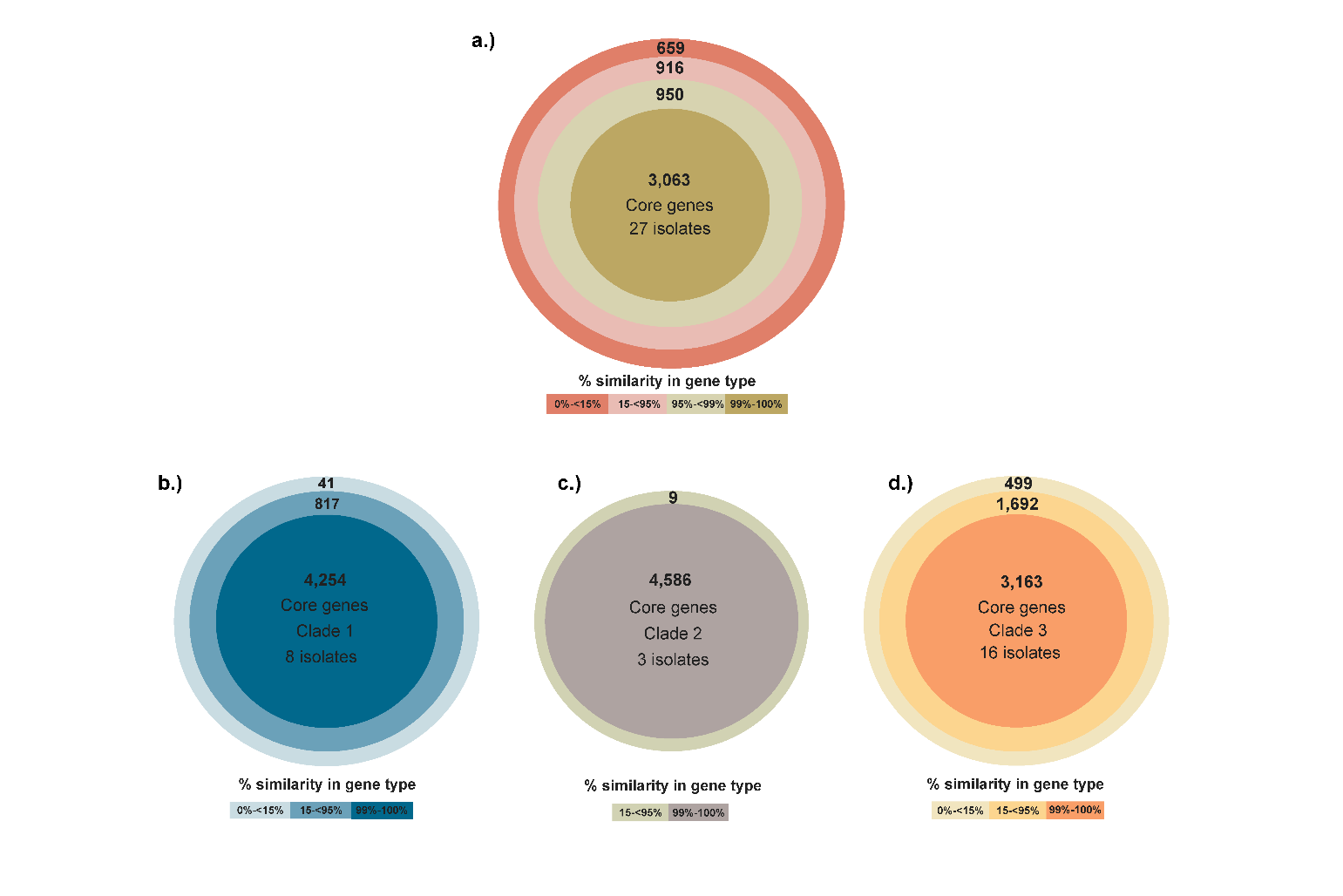
**

**Supplementary Figure 2** Pangenome analysis of the 27 S. 4,[5],12:i:- isolates in this study. The genes were classified based on the percent of homologous genes present in the studied isolates. A gene present in 99%-100% of the isolates was defined as a hard-core gene. A gene present in 95-99% of the isolates was defined as a soft-core gene. A gene present in less than 95% of tested isolates was defined as an accessory gene. a.) Pangenome analysis of 27 S. 4,[5],12:i:- isolates used in this study. With respect to the core genome of the 27 isolates, the number of accessory genes of H1-012, H1-014 and H1-120 were 735, 598 and 691, respectively. The total number of accessory genes found only in our three isolates (H1-012, H1-014, and H1-120) is 58. Fifty-three accessory genes were found in H1-120. Two and four accessory genes were found in H1-014 and H1-012, respectively. b.) Pangenome analysis of isolates in clade 1 c.) Pangenome analysis of isolates in clade 2 d.) Pangenome analysis of isolates in clade 3. Each clade did not contain any soft-core gene.

**
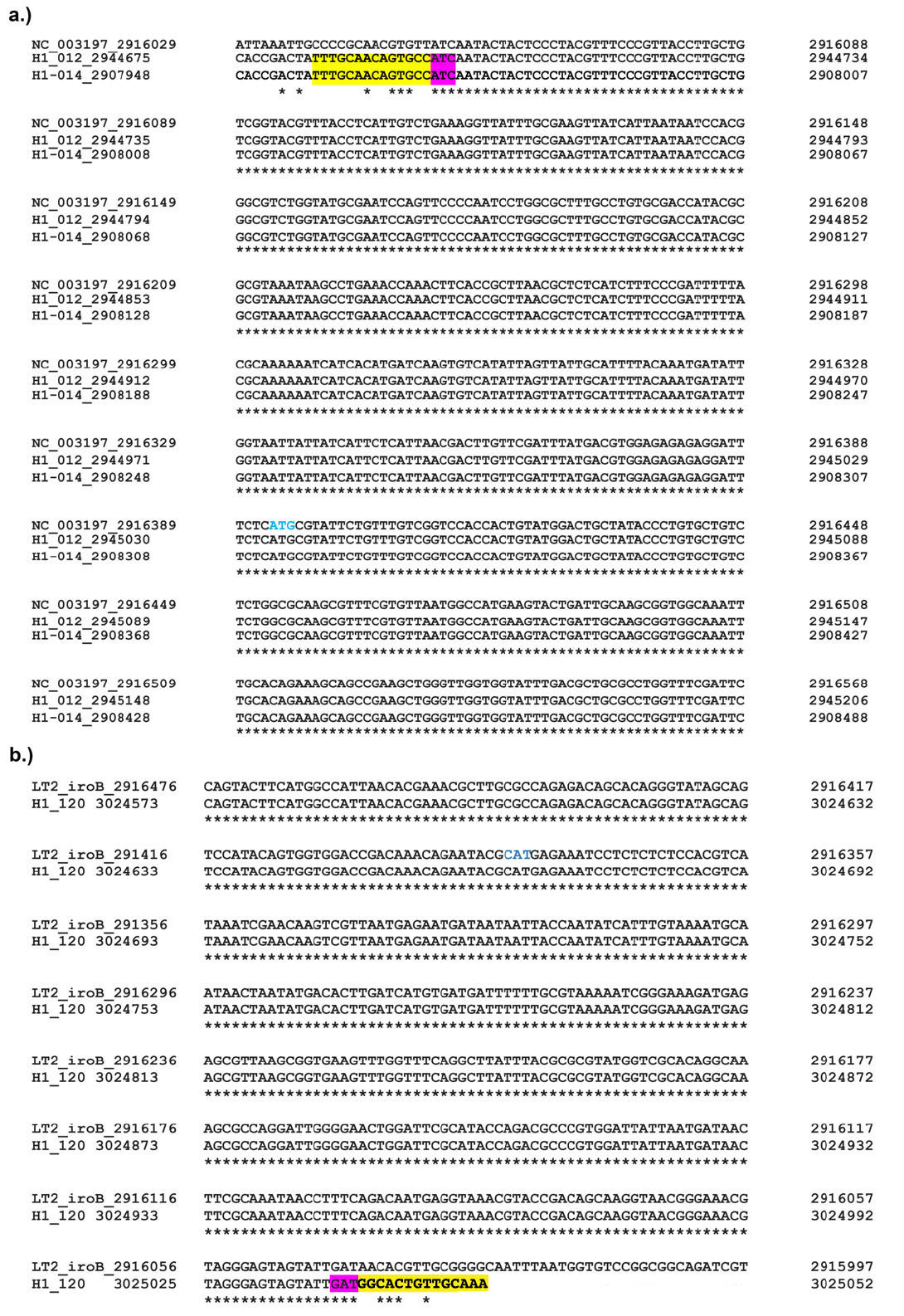
**

**Supplementary Figure 3** Alignment of H1-012, H1-014, and H1-120 in the region near *iroB* using *S.* Typhimurium LT2 as a reference. a.) alignment of H1-012, H1-014 to of LT2 *S.* Typhimurium LT2. b.) alignment of H1-120 to of LT2 *S.* Typhimurium LT2*.* Navy blue letters indicate the start codon of *iroB.* The inverted repeats of IS26 are highlighted in yellow while the pink highlights three adjacent bases, which were the same in all three isolates.

**Supplementary Figure 4** Minimum spanning tree of various alleles of IS*26* in this study and in *Proteus vulgaris* (accession number X00011.1). The cross bars denote the numbers of SNV differences between the most similar pairs of the alleles. This Haplotype network was constructed following Leigh, J. W., & Bryant, D. (2015) ^1^

**Supplementary Table 1** Short read mapping and SNV calling statistics of the 27 *S*. 4,[5],12:i:- isolates with *S.* Typhimurium LT2 as the reference genome

| **Strain** | **Properly-paired mapped reads** | **Total mapped reads** | **Reference genome coverage** | **Average mapping depth: Reference genome-wide** | **Average mapping depth: Mapped region** | **SNVs per isolate** |
| --- | --- | --- | --- | --- | --- | --- |
| 142703 | 1,560,314 (84.56%) | 1,566,414 (84.88%) | 97.78 | 30.57 | 31.26 | 1159 |
| 445956 | 447,050 (92.32%) | 448,343 (92.58%) | 97.28 | 8.21 | 8.44 | 991 |
| 591934 | 3,837,096 (90.73%) | 3,858,757 (91.22%) | 97.80 | 77.39 | 79.13 | 1131 |
| 712472 | 711,660 (83.06%) | 714,719 (83.41%) | 97.43 | 14.54 | 14.93 | 1346 |
| 717816 | 2,132,688 (83.05%) | 2,140,967 (83.36%) | 97.52 | 43.10 | 44.19 | 1576 |
| 782021 | 3,313,638 (86.88%) | 3,331,306 (87.34%) | 97.79 | 67.22 | 68.74 | 1128 |
| 2014AM-2413 | 1,376,528 (91.68%) | 1,388,195 (92.39%) | 97.80 | 52.18 | 53.35 | 1214 |
| A541006 | 192,978 (93.00%) | 193,372 (93.17%) | 95.41 | 6.59 | 6.90 | 1147 |
| A541007 | 236,828 (92.76%) | 237,206 (92.87%) | 96.29 | 7.72 | 8.01 | 1264 |
| A541011 | 258,466 (93.28%) | 258,862 (93.40%) | 95.76 | 8.40 | 8.77 | 1204 |
| A541012 | 253,556 (92.91%) | 254,006 (93.04%) | 95.83 | 8.52 | 8.89 | 1285 |
| A541024 | 354,120 (91.93%) | 354,792 (92.07%) | 97.43 | 12.07 | 12.39 | 1079 |
| A541025 | 266,492 (92.63%) | 266,982 (92.77%) | 97.05 | 9.10 | 9.38 | 1166 |
| A543010 | 169,334 (90.39%) | 169,798 (90.60%) | 95.53 | 5.79 | 6.06 | 1126 |
| AUSMDU00027915 | 673,496 (83.84%) | 674,825 (84.00%) | 97.68 | 16.50 | 16.89 | 1184 |
| CFSAN031364 | 652,930 (91.51%) | 656,654 (91.99%) | 97.76 | 16.98 | 17.37 | 1134 |
| H1-012 | 958,016 (87.90%) | 974,776 (89.21%) | 98.00 | 28.98 | 29.57 | 1110 |
| H1-014 | 1,065,608 (89.14%) | 1,084,627 (90.44%) | 97.71 | 32.29 | 33.05 | 1041 |
| H1-120 | 450,548 (90.89%) | 451,652 (91.1%) | 96.56 | 11.16 | 11.56 | 1271 |
| L-4445 | 772,874 (88.55%) | 784,861 (89.39%) | 97.55 | 25.65 | 26.30 | 1043 |
| L-4567 | 499,510 (88.72%) | 506,176 (89.42%) | 97.54 | 16.78 | 17.20 | 1052 |
| L-4605 | 552,268 (91.97%) | 559,217 (92.64%) | 97.53 | 18.57 | 19.04 | 995 |
| PNUSAS019610 | 1,130,168 (91.33%) | 1,133,870 (91.55%) | 97.50 | 46.28 | 47.46 | 1618 |
| SSI-AC257 | 451,362 (88.29%) | 452,841 (88.53%) | 96.70 | 16.09 | 16.64 | 961 |
| SSI-AC258 | 938,518 (90.76%) | 942,091 (91.05%) | 97.86 | 34.02 | 34.76 | 1160 |
| SSI-AC259 | 548,234 (93.13%) | 549,825 (93.36%) | 97.43 | 19.63 | 20.15 | 1123 |
| SSI-AC261 | 662,542 (88.67%) | 664,428 (88.88%) | 96.98 | 25.65 | 26.45 | 1384 |

**Supplementary Table 2** The long read sequence data statistics and characteristics of the complete genome sequences

| **Data** | **H1-012** | **H1-014** | **H1-120** |
| --- | --- | --- | --- |
| **Total bases (bp)** | 547 M | 1.05 G | 181 M |
| **Read length N50 (bp)** | 18,048 | 23,519 | 21,005 |
| **Number of reads** | 74,239 | 82,961 | 20,717 |
| **Median read length** | 3,483 | 6,915 | 3,557 |
| **Mean read length** | 7,368 | 12,677 | 8,750 |
| **Coverage (x)** | 108 | 213 | 36 |
| **Genome size (bp)** | 5,039,354 | 4,929,877 | 4,991,640 |
| **GC contents (%)** | 52.12 | 52.19 | 52.17 |
| **Plasmid size (bp)** | 180,872 | 180,031 | 222,126 |

**Supplementary Table 3** Pairwise SNV distances of 27 *mcr-3* carrying *S.* 4,[5],12:i:- and *S.* Typhimurium LT2

**Supplementary Table 4** Plasmid information of all isolates in this study.

| **Isolate number** | **Plasmid replicon** | **Plasmid carring *mcr3*** |  |
| --- | --- | --- | --- |
|  |  |  | **mcr Type** |
|  |  |  |  |
| H1-120 | IncHI2, IncHI2A, IncQ1 | IncHI2 (ST3) | *mcr-3.1* |
| H1-012 | IncC | IncA/C (ST3) | *mcr-3.1* |
| H1-014 | IncC, IncQ1 | IncA/C (ST3) | *mcr-3.1* |
| CFSAN031364 | IncQ1 |  | *mcr-3.21* |
| A541025 | IncFII, IncQ1 |  | *mcr-3.1* |
| A541024 | IncFII, IncQ1, Col |  | *mcr-3.1* |
| A541007 | IncFII |  | *mcr-3.1* |
| A541012 | IncFII |  | *mcr-3.1* |
| A541011 | IncFII |  | *mcr-3.1* |
| A541006 | IncFII |  | *mcr-3.1* |
| A543010 | IncHI2, IncQ1 |  | *mcr-3.1* |
| AUSMDU00027915 | IncC, IncQ1 | IncA/C (ST3) | *mcr-3.1* |
| 142703 | IncHI2, IncHI2A, IncQ1 | IncHI2 (ST2) | *mcr-3.11* |
| 445956 | IncFII |  | *mcr-3.1* |
| 591934 | IncC, IncQ1 | IncA/C (ST3) | *mcr-3.1* |
| 712472 | IncC, IncQ1 | IncA/C (ST3) | *mcr-3.1* |
| 717816 | IncC | IncA/C (ST3) | *mcr-3.1* |
| 782021 | IncC, IncQ1 | IncA/C (ST3) | *mcr-3.1* |
| SSI-AC257 | IncC | IncA/C (ST3) | *mcr-3.1* |
| SSI-AC259 | IncFII, IncQ1 | ? | *mcr-3.1* |
| SSI-AC258 | IncC | IncA/C (ST3) | *mcr-3.1* |
| SSI-AC261 | IncFII(pCoo), IncX1 | ? | *mcr-3.20* |
| L-4445 | IncFIB, IncQ1 |  | *mcr-3.1* |
| L-4567 | IncFIB, IncQ1 |  | *mcr-3.1* |
| L-4605 | IncFIB |  | *mcr-3.1* |
| 2014AM-2413 | IncC | IncA/C (ST3) | *mcr-3.1* |

KY924928.1, MG489958, NG055493 and NG060582 are the NCBI accession number of reference sequences of following genes *mcr-3.1, mcr-3.11, mcr-3.20* and *mcr-3.21*, respectively.

**Supplementary Table 5** Pairwise SNV distances between the IS*26* alleles found in this study compared to the one in *Proteus vulgaris*. (accession number X00011.1). This SNV distance table was created following Kumar, 2016 ^2^

|  | **X00011.1_Proteus** | **IS15DI** | **IS26A** | **IS26B** | **IS26C** | **IS26D** | **IS26E** |
| --- | --- | --- | --- | --- | --- | --- | --- |
| **X00011.1_Proteus** | 0 |  |  |  |  |  |  |
| **IS15DI** | 3 | 0 |  |  |  |  |  |
| **IS26A** | 1 | 2 | 0 |  |  |  |  |
| **IS26B** | 2 | 1 | 3 | 0 |  |  |  |
| **IS26C** | 0 | 3 | 1 | 2 | 0 |  |  |
| **IS26D** | 1 | 4 | 2 | 3 | 1 | 0 |  |
| **IS26E** | 4 | 1 | 3 | 2 | 4 | 5 | 0 |

**Supplementary Table 6** AMR genes in each antibiotic class found in H1-012, H1-014 and H1-120 complete genomes as identified by ResFinder^3^.

| Classes | Genes | Location | *Salmonella* | *Salmonella* | *Salmonella* |
| --- | --- | --- | --- | --- | --- |
|  |  |  | H1-120 | H1-012 | H1-014 |
| Aminoglycoside | *aac(6')-Iaa* | C | P | P | P |
|  |  | Pl | No | No | No |
|  | *aac(3)-IId* | C | No | No | P |
|  |  | Pl | No | I | I |
|  | *aph(3')-Ia* | C | No | No | No |
|  |  | Pl | I | P | P |
|  | *aph(3'')-Ib (strA)* | C | P | No | P |
|  |  | Pl | P | P | No |
|  | *aph(6)-Id (strB)* | C | P | No | P |
|  |  | P | P | P | P |
| Aminocyclitol | *aadA1* | C | No | No | No |
|  |  | Pl | P | No | No |
|  | *aadA2* | C | No | No | No |
|  |  | Pl | P | No | No |
| Beta-lactam | *bla_CTX-M-55_* | C | No | No | No |
|  |  | Pl | No | P | P |
|  | *bla_TEM-1B_* | C | P | No | No |
|  |  | Pl | No | P | P |
|  | *bla_TEM-216_* | C | No | No | No |
|  |  | Pl | I | No | No |
| Quinolone | *qnrS1* | C | No | No | No |
|  |  | Pl | No | P | P |
| Folate pathway antagonist | *sul2* | C | P | No | P |
|  |  | Pl | No | P | P |
|  | *sul3* | C | No | No | No |
|  |  | Pl | P | No | No |
|  | *dfrA12* | C | No | No | No |
|  |  | Pl | P | No | No |
| Phenicol | *cat2* | C | No | No | No |
|  |  | Pl | No | I | I |
|  | *floR* | C | No | No | No |
|  |  | Pl | No | F | F |
|  | *cmlA1* | C | No | No | No |
|  |  | Pl | I | No | No |
| Polymyxin | *mcr3.1* | C | No | No | No |
|  |  | Pl | P | P | P |
| Tetracycline | *tetA* | C | No | No | No |
|  |  | Pl | P | P | P |
|  | *tet(B)* | C | I | No | No |
|  |  | Pl | No | No | No |

C = chromosome; Pl = plasmid; P = a gene that has100% identity to the full-length reference sequence; I = a gene that has 100% identity but to shorter length in comparison to the reference sequence; F = a gene that has less than 100% identity and is shorter in comparison to the reference sequence. No = absence of the gene.

**Supplementary Table 7** Comparison between the antibiotic resistance phenotype (AMRP) and the predicted antibiotic resistance phenotype by ResFinder (PAMRP) of in H1-012, H1-014, and H1-120

| Classes | Oorganism | | *Salmonella* | *Salmonella* | *Salmonella* |
| --- | --- | --- | --- | --- | --- |
|  | Strain | | H1-120 | H1-012 | H1-014 |
|  | AMR Phenotype* | | AmpCSTSxT | AmpCtxCCpST | AmpCtxCCpST |
| kBeta-lactam | Ampicillin | Phenotype | R | R | R |
|  |  | ResFinder prediction | 1 | R | R |
|  | Amoxicillin/Clavulanic acid | Phenotype | S | S | S |
|  | Amoxicillin | ResFinder prediction | 1 | R | R |
|  | Amoxicillin+clavulanic acid | ResFinder prediction | S | S | S |
|  | Cefotaxime | Phenotype | S | R | R |
|  |  | ResFinder prediction | S | R | R |
| Amphenicol | Chloramphenicol | Phenotype | R | R | R |
|  |  | ResFinder prediction | 2 | 2 | 2 |
|  | Florfenicol | Phenotype | NA | NA | NA |
|  |  | ResFinder prediction | S | 1 | 1 |
| Quinolone | Ciprofloxacin | Phenotype | S | R | R |
|  |  | ResFinder prediction | S | R | R |
|  | Nalidixic acid | Phenotype | S | S | S |
|  |  | ResFinder prediction | S | S | S |
| Aminoglycoside | Streptomycin | Phenotype | R | R | R |
|  |  | ResFinder prediction | R | R | R |
|  | Gentamicin | Phenotype | Na | NA | NA |
|  |  | ResFinder prediction | S | 2 | 2 |
| Tetracycline | Tetracycline | Phenotype | R | R | R |
|  |  | ResFinder prediction | R | R | R |
|  | Doxycycline | Phenotype | NA | NA | NA |
|  |  | ResFinder prediction | R | R | R |
| Folate pathway antagonist | Sulfamethoxazole/ Trimethoprim | Phenotype | R | **S** | **S** |
|  | Sulfamethoxazole | ResFinder prediction | R | **R** | **R** |
|  | trimethoprim | ResFinder prediction | R | **S** | **S** |
|  | Trimethoprim | Phenotype | NA | NA | NA |
|  |  | ResFinder prediction | R | S | S |
| Polymyxin | Colistin | Phenotype | R | R | R |
|  |  | ResFinder prediction | R | R | R |

R = resistant to antibiotic; S = susceptible to antibiotic; 1 = resistant to antibiotic as predicted by ResFinder but the antibiotic resistance gene is less than 100% identity and shorter in comparison to the reference sequence. 2 = resistant to antibiotic as predicted by ResFinder but the antibiotic resistance gene has a shorter length in comparison to the reference sequence. The sequence was 100% identical to the reference sequence though. *AMR Phenotype is the data from Win, 2021 ^4^

**Supplementary Table** 8 Isolates information and demographic information.

| **SRA accession** | **Strain** | **Country of origin** | **Isolation source** | **Travel to SEA** | **Collection year** | **Additional information** | **Before cleaning** | | **After cleaning** | | **Reference** |
| --- | --- | --- | --- | --- | --- | --- | --- | --- | --- | --- | --- |
|  |  |  |  |  |  |  | **Forward read** | **Reverse read** | **Forward read** | **Reverse read** |  |
|  |  |  |  |  |  |  | **Read number** | **Read number** | **Read number** | **Read number** |  |
| SRR3323016 | 142703 | UK | Human | N/A | 2015 | N/A | 2,129,560 | 2,129,560 | 922,662 | 922,662 | NCBI/Pathogen Detection Database |
| SRR7523681 | 445956 | UK | Human | N/A | 2017 | N/A | 903,101 | 903,101 | 242,125 | 242,125 | Sun,2020 |
| SRR7828444 | 591934 | UK | Human | N/A | 2018 | N/A | 2,814,320 | 2,814,320 | 2,114,652 | 2,114,652 | Sun,2020 |
| SRR8838987 | 712472 | UK | Human | N/A | 2019 | N/A | 770,803 | 770,803 | 428,424 | 428,424 | Sun,2020 |
| SRR8863376 | 717816 | UK | Human | N/A | 2019 | N/A | 1,772,858 | 1,772,858 | 1,284,010 | 1,284,010 | Sun,2020 |
| SRR9922743 | 782021 | UK | Human | N/A | 2019 | Frozen food | 2,569,736 | 2,569,736 | 1,907,012 | 1,907,012 | Sun,2020 |
| SRR4044602 | 2014AM-2413 | USA | N/A | N/A | 2016 | N/A | 1,140,105 | 1,140,105 | 750,762 | 750,762 | Sun,2020 |
| SRR10172181 | A541006 | Thailand | Animal | N/A | 2011 | Swine Feces | 199,786 | 199,786 | 103,753 | 103,753 | Patchanee,2020 |
| SRR10172163 | A541007 | Thailand | Animal | N/A | 2011 | Swine Feces | 216,825 | 216,825 | 127,652 | 127,652 | Patchanee,2020 |
| SRR10172180 | A541011 | Thailand | Animal | N/A | 2011 | Swine Feces | 249,794 | 249,794 | 138,540 | 138,540 | Patchanee,2020 |
| SRR10172179 | A541012 | Thailand | Animal | N/A | 2011 | Swine Feces | 245,223 | 245,223 | 136,453 | 136,453 | Patchanee,2020 |
| SRR10172161 | A541024 | Thailand | Animal | N/A | 2011 | Swine Feces | 323,345 | 323,345 | 192,602 | 192,602 | Patchanee,2020 |
| SRR10172160 | A541025 | Thailand | Animal | N/A | 2011 | Swine Feces | 242,766 | 242,766 | 143,848 | 143,848 | Patchanee,2020 |
| SRR10172193 | A543010 | Thailand | Environment | N/A | 2012 | Swine production environment | 173,569 | 173,569 | 93,665 | 93,665 | Patchanee,2020 |
| SRR14141591 | AUSMDU00027915 | Australia | Human | N/A | 2010 | N/A | 1,540,184 | 1,540,184 | 401,635 | 401,635 | NCBI/Pathogen Detection Database |
| SRR4243014 | CFSAN031364 | Thailand | Animal | N/A | 2013 | Swine Feces | 713,418 | 713,418 | 356,743 | 356,743 | NCBI/Pathogen Detection Database |
| SRR13013442 | H1-012 | Thailand | Human | N/A | 2010 | stool | 1,189,368 | 1,189,368 | 544,932 | 544,932 | This study |
| SRR13013431 | H1-014 | Thailand | Human | N/A | 2010 | stool | 1,285,429 | 1,285,429 | 597,702 | 597,702 | This study |
| SRR8523082 | H1-120 | Thailand | Food | N/A | 2010 | Frozen ready to eat food | 1,086,431 | 1,086,431 | 247,857 | 247,857 | This study |
| DRR236424 | L-4445 | Japan | Animal | N/A | 2014 | Cattle | 1,390,779 | 1,390,779 | 436,417 | 436,417 | Arai,2018 ^5^ |
| DRR236466 | L-4567 | Japan | Animal | N/A | 2014 | Cattle | 854,017 | 854,017 | 281,503 | 281,503 | Arai,2018 ^5^ |
| DRR236500 | L-4605 | Japan | Animal | N/A | 2016 | Cattle | 924,670 | 924,670 | 300,253 | 300,253 | Arai,2018 ^5^ |
| SRR5907352 | PNUSAS019610 | USA | N/A | N/A | 2017 | N/A | 1,033,851 | 1,033,851 | 618,758 | 618,758 | Sun,2020 ^6^ |
| ERR2187914 | SSI-AC257 | Denmark | Human | No | 2010 | N/A | 640,628 | 640,628 | 255,619 | 255,619 | Sun,2020 and ^6^ ^7^ Litrup,2017 |
| ERR2187916 | SSI-AC258 | Denmark | Human | Yes  (Thailand) | 2011 | N/A | 996,314 | 996,314 | 517,009 | 517,009 | Sun,2020 and (2-3)),Litrup,2017 |
| ERR2187915 | SSI-AC259 | Denmark | Human | Yes  (Thailand) | 2011 | N/A | 746,863 | 746,863 | 294,342 | 294,342 | Sun,2020 and Litrup,2017 (2-3) |
| ERR2187918 | SSI-AC261 | Denmark | Human | Yes  (Thailand) | 2016 | N/A | 1,159,896 | 1,159,896 | 373,593 | 373,593 | Sun,2020 and Litrup,2017 (2-3) |
| SRR6060733 | 2008AR-0009 | USA | Human | N/A | 2017 | N/A | 1,020,689 | 1,020,689 | 642,363 | 643,363 | Sun,2020 (2) |

N/A: no applicable

Note: The long read sequencing data of H1-012, H1-014 and H1-120 are available in the Sequence Read Archive repository, under BioProject accession number PRJNA675488 for H1-012 and H1-014 and PRJNA808666 for H1-120

**References**

1 Leigh, J. W. & Bryant, D. popart: full-feature software for haplotype network construction. *Method Ecol. Evol.* **6**, 1110-1116. https://doi:https://doi.org/10.1111/2041-210X.12410 (2015).

2 Kumar, S., Stecher, G. & Tamura, K. MEGA7: Molecular evolutionary genetics analysis version 7.0 for bigger datasets. *Mol. Biol. Evol.* **33**, 1870-1874. https://doi:10.1093/molbev/msw054 (2016).

3 Bortolaia, V. *et al.* ResFinder 4.0 for predictions of phenotypes from genotypes. *J. Antimicrob. Chemother.* **75**, 3491-3500. https://doi:10.1093/jac/dkaa345 (2020).

4 Win, A. T. *et al.* Sequence analyses and phenotypic characterization revealed multidrug resistant gene insertions in the genomic region encompassing phase 2 flagellin encoding *fljAB* genes in monophasic variant *Salmonella enterica* serovar 4,5,12:i:- isolates from various sources in Thailand. *Front. Microbiol.* **12**, 720604. https://doi:10.3389/fmicb.2021.720604 (2021).

5 Arai, N. *et al.* Phylogenetic Characterization of Salmonella enterica Serovar Typhimurium and Its Monophasic Variant Isolated from Food Animals in Japan Revealed Replacement of Major Epidemic Clones in the Last 4 Decades. *J. Clin. Microbiol.* **56**, e01758-01717. https://doi:10.1128/JCM.01758-17 (2018).

6 Sun, R.-Y. *et al.* Global clonal spread of mcr-3-carrying MDR ST34 Salmonella enterica serotype Typhimurium and monophasic 1,4,[5],12:i:− variants from clinical isolates. *J. Antimicrob. Chemother.* **75**, 1756-1765. https://doi:10.1093/jac/dkaa115 (2020).

7 Litrup, E. *et al.* Plasmid-borne colistin resistance gene *mcr-3* in *Salmonella* isolates from human infections, Denmark, 2009–17. *Euro Surveill* **22**, 30587. https://doi.org/10.2807/1560-7917.ES.2017.22.31.30587 (2017).
